# Tennis club governance: an international comparison of Southern European clubs

**DOI:** 10.3389/fspor.2025.1701253

**Published:** 2025-11-18

**Authors:** Francesc Solanellas, Joshua Muñoz, Miguel Crespo, Rafael Martínez-Gallego

**Affiliations:** 1 National Institute of Physical Education of Catalonia (INEFC), University of Barcelona, Barcelona, Spain; 2 Social and Educational Research Group on Physical Activity and Sport (GISEAFE), INEFC, University of Barcelona, Barcelona, Spain; 3 Development Department, International Tennis Federation (ITF), London, United Kingdom; 4 Grupo de Investigación de la Técnica y la Táctica Deportivas, Universitat de València, Valencia, Spain

**Keywords:** racket sports, sports policies, club development, sport organization, management

## Abstract

**Introduction:**

Tennis is one of the world's most practiced racket sports, with clubs serving as the main venues for participation, training, and community engagement. Despite their central role, research on the governance of tennis clubs remains scarce. This study aims to provide an international comparison of governance practices in tennis clubs across Southern Europe.

**Methods:**

The analysis covered 30 tennis clubs from Spain, Italy, Portugal, and Malta. A validated governance assessment model was applied, grounded in three key principles: democracy and participation, ethics and integrity, and accountability and transparency. Key governance indicators included board diversity, stakeholder involvement, president turnover, document transparency, and decision-making structures. Clubs were clustered into four governance types using k-means analysis.

**Results:**

The results revealed marked differences among clubs. Some exhibited balanced and participatory governance, while others demonstrated restricted and opaque practices. Clubs with more independent board members tended to experience greater leadership turnover, and those with higher financial transparency were more likely to disclose governance documents. Conversely, gender equality metrics showed minimal correlation with other governance indicators.

**Discussion and Conclusions:**

The study highlights the need for enhanced inclusivity, structured oversight, and transparency in tennis club governance. The findings offer valuable insights for club managers, federations, and policymakers seeking to professionalize governance in sports organizations. Future research should explore governance variations across club types, regions, and socio-economic contexts, and develop longitudinal strategies for inclusive and sustainable governance practices in tennis.

## Introduction

Tennis is a globally popular sport, engaging around 87 million players and supported by a vast infrastructure that spans over 200 countries and approximately 490,000 courts (1–3). Governed by the International Tennis Federation (ITF), which has 211 member nations, the sport enjoys strong grassroots and elite-level participation. The development and delivery of tennis largely occur within tennis clubs, making them fundamental to the sport's global ecosystem (4, 5). These clubs vary in size, function, and structure, offering essential services such as coaching, competition, and community engagement (6, 7).

Tennis clubs can be broadly categorized into private, public, academy, and corporate clubs (8). Private clubs, often membership-based, provide exclusive services and cater to more affluent demographics (9). Public clubs, typically government-run, focus on accessibility and grassroots development (10, 11). Academy clubs prioritize the competitive development of athletes (12), while corporate clubs aim to enhance employee wellness (13). Services at tennis clubs include coaching, fitness facilities, and opportunities for social interaction (14–16). Clubs serve both casual and competitive players and support holistic development through structured training and community events (17).

With an estimated 45,000 clubs worldwide (2), their governance plays a critical role in shaping the sport. However, governance in tennis clubs has received limited academic attention, despite its implications for accountability, transparency, and ethical leadership. Issues such as board composition, gender equity, stakeholder participation, and financial transparency are central to effective governance.

This study aims to address this gap by offering a comparative analysis of tennis club governance in four European countries: Spain, Italy, Portugal, and Malta. The research draws on core governance principles—democracy and participation, ethics and integrity, and accountability and transparency—as articulated by scholars such as Geeraert et al. (18), and Pielke et al. (19). The goal is to explore how clubs implement these principles in practice and to identify patterns that can inform future improvements in sport governance.

By focusing on clubs—vital organizational units for sport development—this study seeks to contribute to the broader literature on governance in sport, providing empirical insights that may support greater democratization, inclusivity, and strategic planning in tennis organizations worldwide.

## Literature review on tennis club governance and management

Tennis clubs have been studied across a range of themes, including health promotion, psycho-social dynamics, governance structures, and economic sustainability. Despite their significance in the tennis ecosystem, governance remains underexplored.

Tennis clubs contribute significantly to public health and well-being. Dobbinson et al. (20) conducted a cross-sectional study of sports clubs in Victoria, Australia, showing that while many had adopted health promotion policies, their scope and enforcement varied. Similarly, Pluim et al. (13) observed that clubs in the Netherlands that implemented health-promoting activities reported increased member satisfaction and engagement.

Beyond health, the psycho-social environment within tennis clubs has been examined. Carlson (21) investigated the socialization of elite Swedish tennis players, highlighting the importance of early specialization and family support. In Strasbourg, Waser (22) found that social class shaped club dynamics and member interactions. Muir (23) argued that recreational tennis within clubs often reflects professionalized, work-like norms, governed by implicit competitive structures. Falcus and McLeod (24), drawing on Bourdieu's concept of habitus, studied a New Zealand club where youth development was heavily influenced by middle-class values. Lake (25) explored how social exclusion operated within a British tennis club, reinforcing established-outsider distinctions.

Retention and dropout trends have also been a focus of research. Deelen et al. (26) found that youth players who changed schools or played multiple sports were more likely to leave clubs, while participation in social and volunteer activities within clubs promoted retention. Time use was identified as a stronger predictor of dropout than environmental factors, underlining the importance of flexible scheduling and diversified programming.

Inclusion and accessibility have emerged as critical themes in recent literature. Polak-Sopinska and Nebelska (27) examined how clubs adapted to include visually impaired individuals, recommending organizational strategies such as adaptive equipment and staff training. Johnston et al. (28) assessed the female-friendliness of New Zealand's clubs and found that while infrastructure for women was generally adequate, deeper engagement strategies were lacking. Storr and Richards (29) studied tennis's positive impact on LGBT+ participants in Australia, highlighting improved mental health and social cohesion. In South Korea, Lim et al. (30) explored how women navigated club hierarchies and constructed symbolic identities linked to elite group participation.

Tennis clubs also serve as developmental environments for athletes and coaches. Milistetd et al. (31) evaluated a 24-month collaborative program to enhance coach development in a multisport club. Gerdin et al. (32) explored Swedish coaching practices that foster athlete development. In Indonesia, Cakravastia and Setiawan (33) emphasized the role of local clubs in forming the base of the athlete development value chain, stressing the importance of coordinated stakeholder involvement.

Governance, leadership, and strategic management have received growing attention. Valiño (34) outlined key governance issues in tennis clubs, including transparency, board structure, and leadership ethics. Hotta and Yamamoto (35) demonstrated the positive impact of robust governance in Japanese college tennis clubs. Varmus (36) and Varmus et al. (37, 38) studied partnerships between Slovak clubs and schools, revealing the importance of communication strategies and stakeholder alignment. Sotiriadou et al. (39) emphasized the role of inter-organizational collaboration in elite athlete development. Talavera et al. (40) showed that Spanish club managers overwhelmingly valued strategic thinking about stakeholder relations.

Additional contributions include the development of governance tools. Kasale et al. (41) introduced a performance management toolkit for tennis clubs in Botswana. Crespo Dualde (42) proposed the balanced scorecard for performance monitoring and decision-making in club settings. Irigoyen (43) advocated for leadership strategies focused on empowerment and positivity, while Henry (44) provided practical guidance for growing participation and managing aging infrastructure.

The economic dimension of tennis club operations has also been studied. Garrett (45) analyzed the responses of UK clubs to Sport England's Lottery funding, documenting varied patterns of compliance and resistance. Abdel (46) identified key financial returns of tennis in Egyptian private clubs, such as revenue from lessons, facility rentals, and merchandising. Kellett (47) compared volunteer-run and commercially managed clubs in Australia, noting that the latter were more strategic in reserve allocation, investing in programs rather than solely in infrastructure. Morrison and Misener (48) highlighted strategic planning and effective resource management as key to club success.

Marketing and member satisfaction are also crucial themes. Kölbl et al. (49) developed a comprehensive Member Satisfaction Index (MSI) tested in a major German club, identifying drivers of member retention. Schulz et al. (50) segmented clubs in a regional tennis federation into expectation-based types, helping tailor services and policies. Marketing strategies were addressed by Kellett and Fielding (51) in their analysis of the Louisville Racquet Club. Simozima et al. (52) classified club members by motivation, forming six distinct participant clusters based on goals like skill mastery, fitness, and recognition.

The structural diversity of tennis clubs also affects governance and service provision. Simojima and Kimura (53) evaluated junior programs in commercial clubs in Japan, identifying benefits like brand enhancement and coach development, but also highlighting drawbacks such as financial strain and member dissatisfaction. Peric and Wise (54) examined two tourist-focused clubs in Croatia and concluded that user experience was driven more by court quality than by organizational models. Riba (7) noted a growing trend of outsourcing club management to sports companies, enhancing service delivery and professionalization. In Thailand, Panjasilpa (55) developed a business model for tennis training centers, identifying critical factors like service quality, satisfaction, and loyalty.

Crisis adaptation and innovation have gained importance since the COVID-19 pandemic. Crespo Celda et al. (56) and Crespo et al. (16) studied Latin American federations and clubs, highlighting the implementation of digital solutions such as virtual coaching and online engagement. Tinaz and Emiroglu (57) offered a reopening strategy for tennis clubs based on SWOT analysis, calling for clubs to adapt to shifting consumer behavior post-pandemic.

Social capital and the role of volunteers form another essential area of research. Hallmann and Dickson (58) found that club size and working hours influenced volunteer availability in New Zealand. Board and committee roles, as well as the presence of junior members, increased volunteer engagement. Tacon (59) explored the formation of social ties in UK clubs, showing how voluntary sports organizations foster both strong and weak ties among members. He emphasized the importance of organizational context in cultivating trust, cooperation, and social cohesion.

Together, these studies provide a multi-dimensional understanding of tennis club functioning and development. While substantial progress has been made in exploring inclusion, health, coaching, and economic management, there remains a notable gap in empirical and comparative research on governance practices. The evidence suggests that clubs with structured oversight, stakeholder participation, financial transparency, and strategic vision are more sustainable. However, more longitudinal and cross-cultural studies are needed to explore how governance reform can lead to long-term improvements in equity, engagement, and performance across diverse tennis club environments.

## Methods

### Instrument

Examining the principles of good governance in sports often highlights the absence of a unified and standardized framework (18). This study focuses on evaluating three critical aspects deemed essential for good governance within sport organizations: democracy and participation, ethical conduct and integrity, as well as transparency and accountability (18, 19). To accomplish this, a bespoke assessment model was designed, drawing methodological insights from previous works on scale development and composite indicators (60–62). The model also builds on approaches applied by Muñoz et al. (63) in their analysis of governance within Catalan Sport Federations. The creation of this framework adhered to a meticulous, phased methodology:

a. Development of measurement items:
  - Conceptual framework and indicators design: A multidimensional model was conceptualized, targeting specific governance objectives through quantitative indicators capable of evaluating practices within the identified dimensions. These indicators were derived from a comprehensive literature review, encompassing key contributions on governance best practices [e.g., (64–68), among others].
  - Indicators validation: The initial set of indicators underwent a rigorous validation process involving 15 subject-matter experts, including both practitioners and academics.
b. Construction of the scale:
  - Normalization protocols: To address the diverse units of measurement across indicators, all data were normalized. Scores were first expressed as percentages and subsequently converted to a unified scale ranging from 0 to 10.
  - Weight assignment for indicators: A survey was administered to 15 experts (comprising general secretaries of sport clubs and federations and professionals experienced in sport performance metrics) to determine the relative importance of each indicator. Using a 0 (no importance) to 5 (highest importance) rating system, the average scores from these evaluations were employed to calculate the proportional weight of each indicator within its respective dimension.
c. Evaluation of the scale:
  - System reliability check: The internal consistency of the measurement framework was assessed using the Cronbach's alpha coefficient to ensure robustness.

Table 1 provides an overview of the justification behind the selection of indicators included in the governance measurement framework. It also outlines the specifics of the measurement scale and highlights the assigned relative weight of each indicator within its respective dimension.

**Table 1:** Framework implemented for measuring governance in sport organisations.

Dimension	Objective	Indicator	Rationale	Measurement scale
Democracy and participation (Weight: 30%, *α* = 0.504)	Enhance democracy and stakeholder engagement	Number of organizational committees	Diverse committees (e.g., executive, financial, ethics, etc.) foster participative decision-making by including multiple stakeholders	Discrete numerical. Committees present/10.
		Holding general assemblies	Annual assemblies are essential for accountability, as they allow members to evaluate the leadership’s performance	Dichotomous (Yes = 10, No = 0).
		Representation in the general assembly	Greater representation from various groups (e.g., clubs, athletes) enhances inclusiveness and democratic functioning	Discrete numerical. Representatives/5.
Ethics and integrity (Weight: 41%, *α* = 0.456)	Strengthen organizational ethical standards and integrity	Gender diversity on the board	Promoting women in leadership roles reflects commitment to equity and ethical responsibility	Discrete numerical. Women/Total. (+20% range). Scored from 0 to 10.
		Presence of independent board members	Independent members reduce bias and strengthen impartial decision-making	Discrete numerical. Independent members. (0 = 0; 1 = 8; >1 = 10).
		Presidential term turnover	Regular board turnover prevents power monopolies and promotes organizational renewal	Discrete numerical. Average years scored from 0 to 10 based on tenure.
		Term limits and length of presidency	Mandates with limits ensure transparency and reduce governance risks associated with prolonged tenures	Discrete numerical. Length scored within 20% ranges (e.g., <8 = 10).
Accountability and transparency (Weight: 28%, *α* = 0.616)	Promote organizational responsibility and transparency	Availability of governance documents	Documents like codes of ethics or governance policies ensure accountability and provide operational clarity	Discrete numerical. Number of documents/14.
		Distribution of financial results	Publishing financial information in a timely manner builds trust and transparency with stakeholders	Dichotomous (Yes = 10, No = 0).
		Disclosure of activity reports	Publicly available documents on organizational actions increase transparency and stakeholder confidence	Discrete numerical. Documents published/14.

### Sample

This study adopts an international comparative approach to explore governance practices within tennis clubs. Four countries (Spain, Italy, Portugal, and Malta) were selected for their relevance to the researchers and their participation in the Erasmus+ project, *Open Data For Sport Governance*. This initiative aims to promote transparency and good governance in sport across Europe by fostering collaboration among sport entities in these nations. The selected countries share commonalities in their sport systems, including a predominance of member-based governance structures, substantial reliance on public funding, and the use of clubs as foundational units for sports development.

To ensure a comprehensive analysis, we preselected 48 tennis clubs across the four countries, targeting a range of club sizes. Clubs were stratified by membership size, with the aim of representing diverse operational scales: 2 clubs per country with fewer than 100 members, 2 with 100–200 members, 2 with 201–300 members, 2 with 301–600 members, 2 with 601–1,800 members, and 2 exceeding 1,800 members. The average membership size of the preselected clubs was 853.37 ± 1,185.5.

The response rate for participation was 62.5%, yielding a final sample of 30 tennis clubs. These clubs were distributed as follows: Spain (10 clubs), Italy (7 clubs), Portugal (8 clubs), and Malta (5 clubs). Table 2 provides an overview of the final sample, highlighting its representativeness across the predefined membership categories. This diverse and internationally distributed sample enables a meaningful exploration of governance practices in tennis clubs across different territorial and organizational contexts.

**Table 2:** Final sample of tennis clubs by country and membership size.

Country	<100 members	100–200 members	201–300 members	301–600 members	601–1,800 members	>1,801 members	Total	%
Italy	4	1	1	1			7	**23%**
Malta		2	2	1			5	**17%**
Portugal	2	1	3	1	1		8	**27%**
Spain		1	1		3	5	10	**33%**
**Total**	**6**	**5**	**7**	**3**	**4**	**5**	**30**	**100%**
**%**	**20%**	**17%**	**23%**	**10%**	**13%**	**17%**	**100%**	

Bold values indicate the percentage within each column/row. Percentages are calculated relative to the sample size for each country or club size category.

Through the invitation emails, organisations' president and general secretary were informed about the research project aim. In addition, online meetings were scheduled to discuss the project in more detail, as well as to resolve possible doubts about the questionnaire. The emails contained a personalised link to the online questionnaire that allowed respondents to log in and log out while completing the data. Respondents were required to complete the questionnaire based on the practices of their organisations and were asked to provide data in reference to the year 2019, the year before the questionnaire was administered because it was the latest household year completed.

### Procedure

Data collection relied on two primary sources:

- Secondary Data: The information was compiled from publicly available reports available on the clubs’ official websites.
- Primary Data: A questionnaire was designed and underwent a thorough validation process. Initially, the draft questionnaire was reviewed by 15 field experts, whose feedback informed revisions before conducting a pilot test. The pilot was carried out with 10 sport organizations not included in the main study, to assess the questionnaire's clarity and completion time. Insights from both the expert review and the pilot test were instrumental in finalizing the version of the questionnaire used in the study.

### Data analysis

The data analysis was conducted using RStudio Version 2024.04.1 + 748 for MAC. The first step involved cleaning the dataset and detecting any anomalies to ensure the data was prepared for analysis. This included handling missing values, identifying outliers, and ensuring consistency across the dataset. The analysis started by extracting the relevant performance indicators (KPIs) from the dataset. To ensure comparability across variables, normalization was applied to all KPIs, transforming them to a common scale.

The elbow method was employed to determine the optimal number of clusters by analyzing the total within-cluster sum of squares (WSS), which is the sum of squared distances between each data point in a cluster and the cluster centroid (69). By plotting the number of clusters (K) against the WSS values, a clear “elbow” point is expected, where a significant reduction in WSS is observed as the true number of clusters is approached, followed by a more gradual decrease thereafter (69). Subsequently, a k-means clustering algorithm was applied with the predefined four-cluster solution. This method partitioned the data into distinct clusters, each representing a group of tennis clubs with similar governance characteristics as defined by the KPIs. The assigned cluster for each data point was then added back to the original dataset for further analysis.

To assess the significance of differences in KPI values between clusters, an ANOVA was conducted for each KPI. *Post-hoc* comparisons were performed using Bonferroni correction to account for multiple comparisons. Effect sizes were calculated and reported using eta squared (*η*^2^), which provides a measure of the proportion of variance in the KPI explained by the clustering. An *η*^2^ value of 0.01 was interpreted as a small effect, 0.06 as a medium effect, and 0.14 or greater as a large effect. Additionally, correlation analysis was performed on the renamed KPIs to investigate potential relationships between the variables. The analysis used nonparametric Spearman's correlation coefficients. The significance level for all the analysis was set at *p* < 0.05.

To explore the underlying structure of the KPIs and their relationships across clusters, radar charts were generated for each cluster. These visualizations highlighted the average performance within each cluster across the different KPIs, facilitating a comparative analysis between clusters.

## Results

The clustering analysis revealed distinct groupings of tennis clubs based on organizational and governance indicators. Cluster 2 emerged as the largest, comprising 16 clubs, with a notable concentration from Spain and Portugal (8 and 5 clubs respectively). This suggests a possible regional alignment in club governance practices within these countries. Cluster 1 and Cluster 3 were of equal size (6 clubs each), with Cluster 1 including clubs from Italy, Malta, and Portugal, and Cluster 3 showing a more balanced distribution among Malta, Portugal, and Spain. Cluster 4, the smallest, included only two clubs, both from Italy, indicating a potentially unique profile or outlier behaviour. Notably, Spanish clubs appear predominantly in Cluster 2, highlighting a pattern that could reflect similar governance structures or organizational characteristics among these institutions.

Table 3 shows the comparison of governance metrics across the four identified clusters of tennis clubs. Regarding the number of members, Cluster 2 had the largest average (1,283.44 ± 1,422.92), while Cluster 4 had the smallest (24.50 ± 0.71), though the effect size was small (*η*^2^ = 0.171). The number of committees differed more substantially between clusters, with Cluster 3 having the highest average number of committees (4.83 ± 1.72), significantly higher than Clusters 1, 2, and 4 (*η*^2^ = 0.613).

**Table 3:** Comparison of governance metrics across tennis club clusters.

Dimension	Indicator	Cluster 1	Cluster 2	Cluster 3	Cluster 4	*η* ^2^
Size	Number of members	243.33 (162.69)	1283.44 (1,422.92)	592.83 (809.98)	24.50 (0.71)	0.171
Democracy and participation	Committees	1.33 (0.52)^@^	1.69 (1.08)^@^	4.83 (1.72)^*^^,^^$^^,^^#^	1.00 (0.00)^@^	0.613
	General assembly	10.00 (0.00)^#^	9.38 (2.50)^#^	10.00 (0.00)^#^	0.00 (0.00)^*^^,^^$^^,^^@^	0.653
	Meetings advisory committee	0.67 (0.82)	1.13 (0.62)	1.17 (0.75)	0.00 (0.00)	0.204
Ethics and integrity	Gender equality	5.67 (4.46)	2.63 (2.71)	5.00 (3.29)	0.00 (0.00)	0.230
	Independent members	10.00 (0.00)^$^^,^^@^	0.63 (2.50)^*^^,^^#^	0.00 (0.00)^*^^,^^#^	9.00 (1.41)^$^^,^^@^	0.847
	President turnover	3.33 (5.16)^$^	8.44 (3.52)^*^^,^^@^^,^^#^	0.00 (0.00)^$^	0.00 (0.00)^$^	0.559
	Mandates length	9.67 (0.82)	9.50 (1.55)	7.33 (4.32)	10.00 (0.00)	0.157
Accountability and transparency	Financial results distribution	8.33 (4.08)	8.75 (3.42)^#^	6.67 (5.16)	0.00 (0.00)^$^	0.270
	Number of documents	4.33 (3.98)^$^	12.25 (5.15)^*^^,^^#^	11.17 (3.71)	1.00 (0.00)^$^	0.447
	Publicly disclose documents	2.00 (3.16)	6.88 (5.61)	3.33 (2.94)	0.00 (0.00)	0.236

Mean (SD).

*η*^2^: eta squared.

* Significant differences to Cluster 1.

$ Significant differences to Cluster 2.

@ Significant differences to Cluster 3.

# Significant differences to Cluster 4.

In terms of democracy and participation, significant differences were found in the functioning of the general assembly. Clusters 1 and 3 reported complete participation (10.00 ± 0.00), whereas Cluster 4 had no participation (0.00 ± 0.00), with a moderate effect size (*η*^2^ = 0.653). Differences in the number of meetings of advisory committees were smaller (*η*^2^ = 0.204), with the highest average found in Cluster 3 (1.17 ± 0.75). Gender equality did not show significant differences between clusters, with a small effect size (*η*^2^ = 0.230).

Ethics and integrity metrics, specifically the presence of independent members on the board, varied significantly between clusters (*η*^2^ = 0.847). Cluster 4 had the highest average (9.00 ± 1.41), while Clusters 1 and 3 reported no independent members (0.00 ± 0.00). President turnover also showed moderate differences across clusters (*η*^2^ = 0.559), with Cluster 2 reporting the highest turnover (8.44 ± 3.52), significantly higher than Clusters 1, 3, and 4. In contrast, no significant differences were observed in the length of mandates, with a small effect size (*η*^2^ = 0.157).

Accountability and transparency metrics showed smaller differences. Financial results distribution had a small effect size (*η*^2^ = 0.270), with the most transparent distribution observed in Clusters 1 (8.33 ± 4.08) and 2 (8.75 ± 3.42). However, the number of documents publicly disclosed varied more significantly (*η*^2^ = 0.447). Clusters 2 and 3 had the highest averages (12.25 ± 5.15 and 11.17 ± 3.71, respectively), significantly higher than Cluster 4 (1.00 ± 0.00), with Cluster 1 also disclosing significantly fewer documents than Cluster 2.

Overall, these results illustrate substantial variation in governance practices across tennis clubs. Some metrics, such as the presence of independent members and president turnover, showed moderate to large differences between clusters, while others, such as financial transparency, revealed smaller yet meaningful differences.

These results not only distinguish the structural diversity of governance practices among clubs but also reveal the lived implications of such differences. Clubs with higher participatory mechanisms and document disclosure tend to cultivate greater trust and shared responsibility, while those with limited participation or opaque procedures often reproduce hierarchical decision-making cultures. In this sense, governance metrics mirror patterns of inclusion, communication, and mutual confidence that define everyday organizational life.

**Figure 1:**
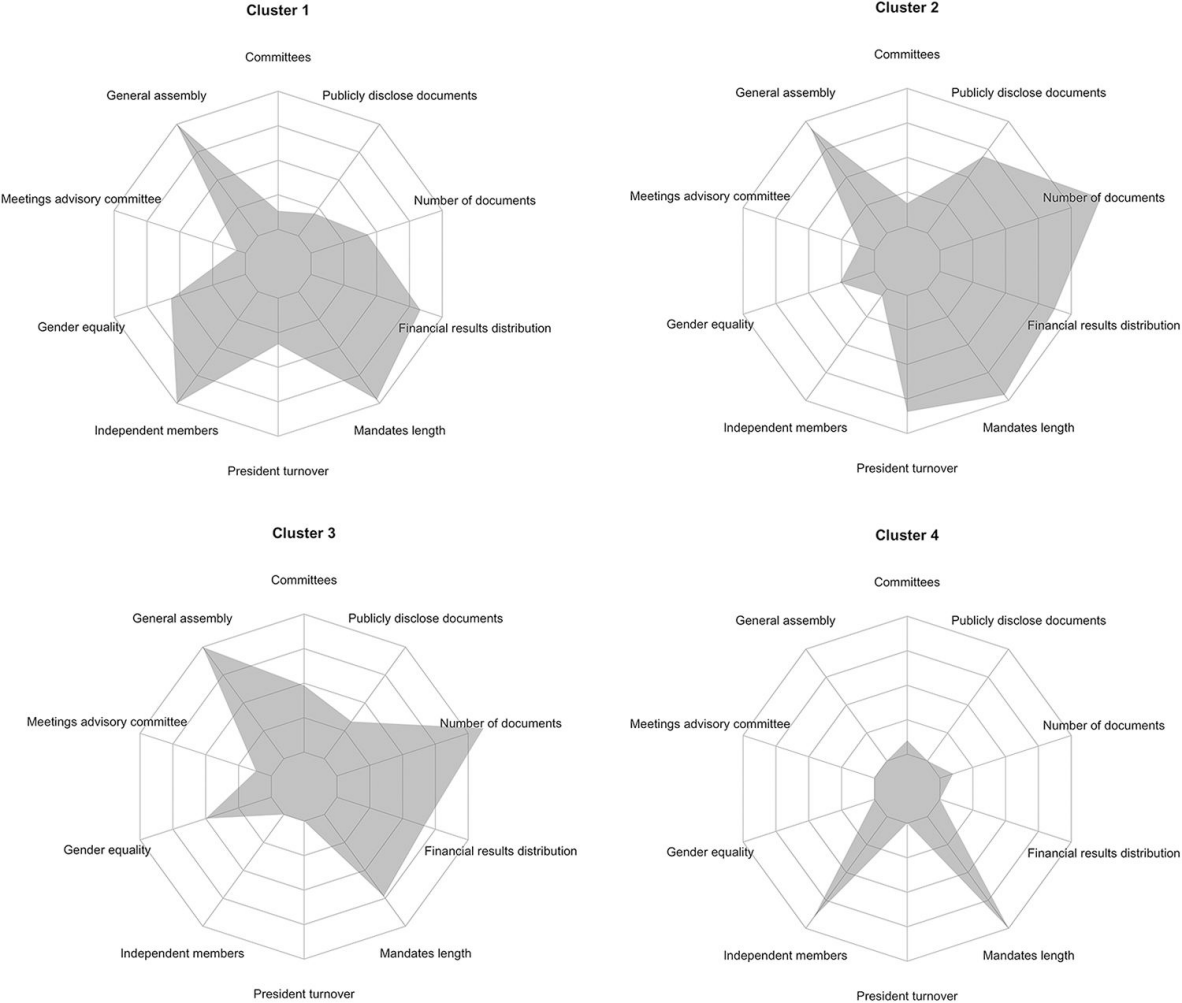
Governance metric distribution across clusters.

Figure 1 illustrates the governance characteristics of the four types of tennis clubs identified through the cluster analysis. Each radar chart represents the distribution of key performance indicators (KPIs) across the clusters, providing a visual summary of the governance structures in each group. The governance metrics include committees, general assembly participation, advisory committee meetings, gender equality, independent members, president turnover, mandate length, financial results distribution, number of documents, and publicly disclosed documents.

Cluster 1—Balanced governance clubs—shows a relatively balanced governance profile, characterized by high scores in mandate length and gender equality. The number of committees and documents publicly disclosed are moderate, while the presence of independent members and advisory committee meetings is limited compared to other clusters.

Cluster 2—Transparent but unstable clubs—is marked by a more uneven governance structure. This cluster has the highest values in financial results distribution and document disclosure but shows lower values in gender equality and independent members. Notably, president turnover is significantly higher, indicating potential governance instability.

Cluster 3—Engaged and well-structured clubs—displays a well-rounded governance profile with moderate to high scores across most metrics. This cluster stands out for its higher-than-average number of committees and strong participation in the general assembly, while gender equality and financial results distribution remain comparable to the other clusters.

Cluster 4—Restricted governance clubs—demonstrates the most constrained governance characteristics, with minimal participation in the general assembly, absence of independent members, and very limited public disclosure of documents. Despite these limitations, the mandate length remains consistent with the other clusters.

These radar charts provide a clear representation of the governance features that distinguish each of the four types of tennis clubs. The variation in governance practices—ranging from well-balanced structures in Clusters 1 and 3 to more limited practices in Cluster 4—reflects the diverse approaches to accountability, transparency, and participation within the different clusters.

The radar charts illustrate not only statistical contrasts but also distinct governance “personalities.” Balanced and engaged clubs (Clusters 1 and 3) reflect cultures of shared leadership and deliberation, where decision-making is perceived as a collective endeavour. In contrast, restricted clubs (Cluster 4) portray governance as concentrated and less dialogic, often limiting opportunities for member voice. These profiles underscore that governance structures are not merely technical arrangements but expressions of how communities choose to organize power, responsibility, and belonging.

**Figure 2:**
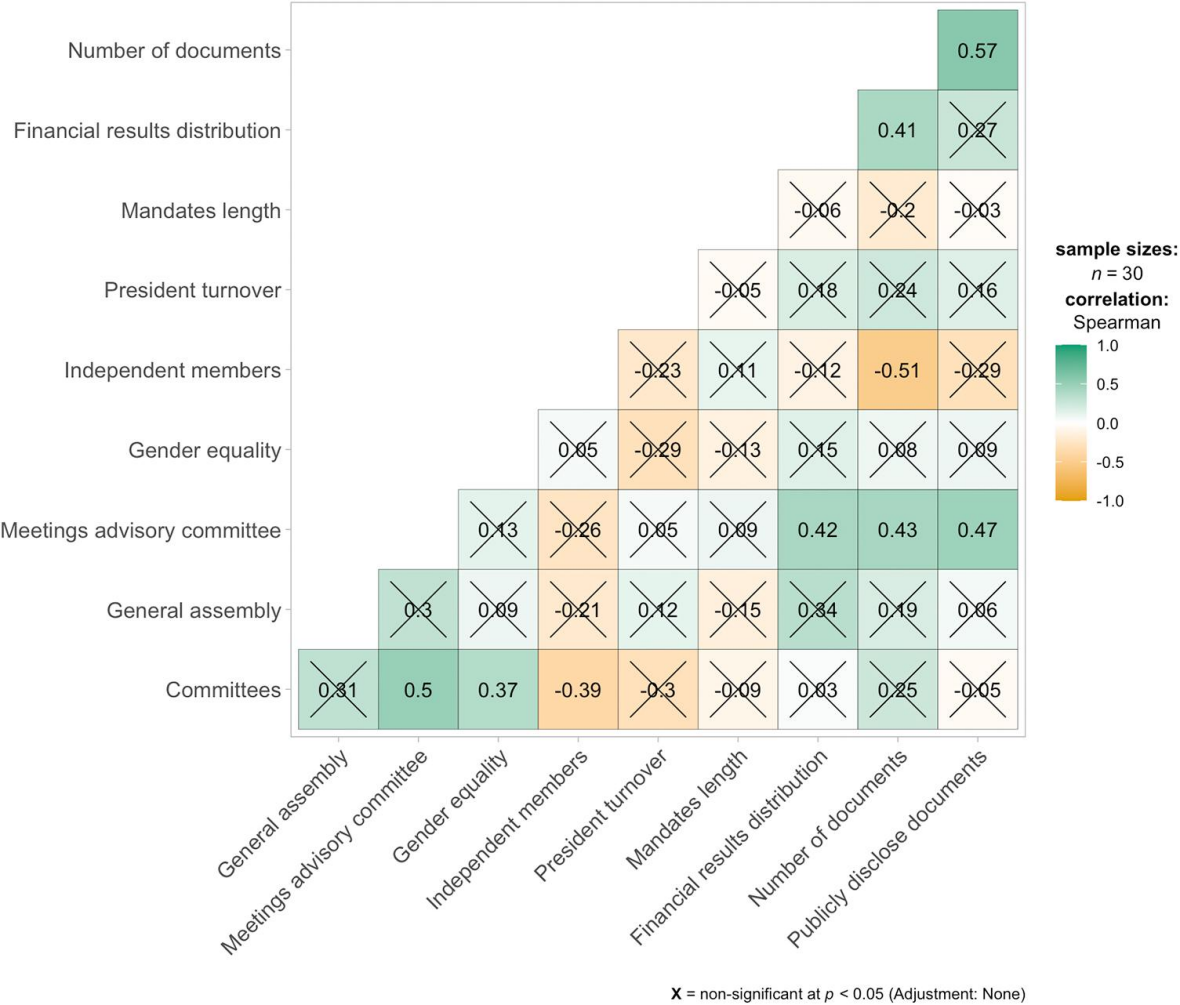
Correlation matrix of tennis clubs’ governance metrics.

Figure 2 shows the correlation matrix for the governance metrics analysed across tennis clubs. Several significant positive correlations were observed between the governance metrics. Notably, the number of documents disclosed was strongly correlated with financial results distribution (*ρ* = 0.67), suggesting that clubs with more transparent financial practices are also more likely to publicly disclose governance-related documents. Similarly, mandate length and financial results distribution were positively correlated (*ρ* = 0.47), indicating that clubs with better mandate scores tend to have more transparent financial distributions.

In terms of leadership stability, a significant positive correlation was found between president turnover and independent members (*ρ* = 0.57). This implies that clubs with more independent members on their boards tend to experience higher turnover rates in the presidency. Conversely, president turnover was negatively correlated with committees (*ρ* = −0.39), indicating that clubs with more committees tend to have more stable presidencies.

Additionally, committees and general assembly participation were positively correlated (*ρ* = 0.43), suggesting that clubs with more formalized committees also show higher engagement in general assembly meetings. However, gender equality did not show strong or significant correlations with most of the governance metrics, indicating that its relationship with other governance practices may be more complex or less direct.

Overall, this correlation matrix highlights several important relationships between governance metrics, particularly in areas related to transparency, leadership turnover, and organizational structure. The strongest associations were observed in metrics involving document disclosure and financial transparency, while other governance aspects, such as gender equality and advisory committee meetings, did not display strong correlations with other variables.

The correlation patterns suggest that transparency and independence act as relational anchors within clubs. When documents and financial information are shared openly, trust expands, and members are more likely to perceive governance as fair and responsive. Conversely, weak correlations between gender equality and other indicators highlight that equity cannot be assumed to emerge automatically from transparency or participation; it requires intentional cultural work and sustained commitment.

## Discussion

This study examined governance practices in tennis clubs across four countries, providing insights into democracy and participation, ethics and integrity, and accountability and transparency. The findings reveal significant variation across governance clusters, highlighting both strengths and areas for improvement.

These governance dimensions—democracy, ethics, and accountability—should be interpreted not only as analytical categories but as expressions of shared human values such as trust, fairness, and mutual respect. This framing emphasizes the relational and ethical nature of governance within tennis organizations. The findings reveal key dynamics regarding participation, distribution of power, and ethical culture, reflecting the tension between community-based governance traditions and modern professional management expectations in Southern Europe.

### Democracy and participation

The findings, consistent with prior studies [e.g., (18, 19, 63)], underscore the critical importance of stakeholder representation in decision-making processes. Among the sampled tennis clubs, significant variability was observed in the inclusivity and participatory mechanisms employed. While general assemblies were consistently present, their composition often failed to adequately reflect the diversity of key stakeholders, such as players, coaches, and external partners.

In this sense, democracy within sport governance is not merely a procedural requirement but a question of voice and inclusion—the extent to which different groups are heard, valued, and able to influence decisions that affect them. It reflects both the fairness of participation and the emotional climate of belonging within the organization.

The analysis revealed notable differences in participation within general assemblies. Clusters 1 and 3 exhibited full stakeholder engagement, whereas Cluster 4 showed no participation, highlighting disparities in democratic governance. Clubs with higher levels of engagement in general assemblies demonstrated more robust governance structures, underscoring the value of fostering inclusive participation mechanisms. In contrast, differences in advisory committee activity were smaller, with Cluster 3 showing slightly more frequent meetings. This suggests that while advisory committees contribute to governance, general assembly participation plays a more pivotal role in fostering democratic practices.

Despite these variations, gender equality metrics were consistent across clusters, revealing a persistent challenge that remains unlinked to other governance practices. As Mulgan (70) and Geeraert et al. (18) argues, democratic governance requires mechanisms enabling stakeholders to hold leadership accountable (a principle unevenly applied across the clubs studied). These findings highlight the need for tailored strategies to enhance stakeholder representativeness and amplify diverse voices in tennis club governance.

Viewed through the lens of power, participation reflects not only who is present in decision-making spaces but whose voices shape outcomes. Clubs with stronger participatory mechanisms distribute authority more horizontally, fostering cultures of dialogue and collective agency. Conversely, limited participation often coincides with concentrated power and lower levels of perceived legitimacy among members.

### Ethics and integrity

Board diversity and gender representation emerged as critical governance aspects across the international sample, reflecting consistent challenges highlighted in previous research (71–73). The inclusion of women in decision-making roles remained limited, signalling significant room for improvement. Given the well-documented benefits of diverse boards (ranging from more ethical decision-making to enhanced organizational performance) [i.e., (74)] tennis clubs must prioritize strategies to recruit and retain women in leadership roles.

Another critical area of concern was the integration of independent board members. The presence of these members varied significantly, with Cluster 4 “Restricted governance clubs” reporting the highest average, while Clusters 1 and 3 reported none. Although this may initially seem counterintuitive, the findings align with the observed positive correlation between independent board members and higher president turnover in Cluster 2. This relationship suggests that independent members can act as governance checks and balances, potentially driving leadership changes and promoting accountability.

Leadership turnover and term limits, indicators of power distribution, also varied across clusters. High president turnover in Cluster 2 highlighted potential governance instability, whereas consistent mandate lengths across clusters suggested that tenure alone might not be a significant driver of governance differences. While leadership stability can promote continuity, unchecked concentration of power risks stifling innovation and accountability (75).

These findings point to opportunities for adopting best practices from higher-performing federations or organizations that have successfully diversified their governance structures (64). Emphasizing gender diversity, external perspectives, and balanced leadership structures can help tennis clubs achieve more robust and inclusive governance (63).

Ultimately, ethics in sport governance goes beyond compliance and formal oversight—it represents the invisible fabric of honesty, care, and responsibility that holds communities together. It is expressed through everyday acts of trust, empathy, and fairness that sustain collaboration within clubs. Understanding ethics in this way allows governance to be seen not merely as a managerial function but as a moral and relational practice that binds organizations to their values and to the people they serve.

### Accountability and transparency

Accountability mechanisms varied significantly among the sampled clubs, with larger organizations generally performing better in this dimension. This disparity may stem from larger clubs' greater capacity to develop formal governance structures. Nevertheless, a common issue across all contexts was the widespread absence of essential governance documents, including strategic plans, ethics codes, and transparency reports. As Bovens (76) and Pielke et al. (19) noted, the lack of such documentation undermines stakeholders' ability to hold organizations accountable, increasing the risk of corruption and mismanagement.

Transparency, closely tied to accountability, was similarly inconsistent. Clusters 2 and 3 stood out for their relatively high levels of document disclosure, aligning with a strong positive correlation between document disclosure and financial results distribution. This suggests that clubs prioritizing financial transparency are also proactive in sharing governance-related information. In contrast, Cluster 4 disclosed minimal documentation, reinforcing its characterization as having restricted governance practices. These disparities underscore the urgent need for systemic efforts to standardize transparency practices across all sport organizations, as emphasized by Aucoin and Heintzman (77).

Beyond formal reporting, transparency in sport governance is ultimately about trust. When information becomes a bridge rather than a barrier, it connects leaders and members through shared understanding and accountability. Genuine transparency transforms data into dialogue—it invites participation, reduces suspicion, and affirms that governance is a collective rather than a closed process. In this sense, trust is not a by-product of transparency but its very foundation.

Correlation analysis revealed additional insights into the interplay between transparency, leadership stability, and organizational structure. Clubs with more formal committees tended to exhibit greater stability in presidencies and higher levels of participation in general assemblies, highlighting the importance of organizational structures in fostering stable and participatory governance. However, gender equality metrics showed weak correlations with other governance indicators, suggesting that achieving gender balance requires targeted initiatives beyond broad structural reforms.

A cross-country comparison highlighted further discrepancies. While systems in Spain and Portugal have begun to implement transparency measures, others, particularly in Malta and Italy, lag behind. Addressing these gaps will require coordinated international efforts to promote standardized accountability and transparency practices across all governance contexts.

These contrasts reveal that governance is not only shaped by regulation and structure but also by culture—by how communities understand responsibility, leadership, and collective purpose. The Southern European clubs studied here embody a complex tension between traditional, community-based governance rooted in volunteerism and belonging, and the modern expectations of professionalism, documentation, and formal oversight. This coexistence creates both richness and friction, suggesting that governance reforms must respect local identities while advancing ethical and transparent practices.

Moving from description to reflection, it becomes clear that the study of governance in sport cannot be confined to procedures and metrics alone. To understand governance is to understand relationships—how trust is built, how power is shared, and how institutions care for their members. In this sense, the humanity of sport organizations is not a limitation to be corrected but a resource to be nurtured, linking accountability with empathy, and structure with meaning.

## Conclusion

This study provides a comprehensive understanding of governance diversity in tennis clubs within an international comparative framework. By identifying key differences in governance practices across clusters and highlighting areas of strength and weakness across four countries, it underscores the interplay between democratic participation, ethical governance, and accountability. The findings offer actionable insights for practitioners and policymakers, emphasizing the importance of fostering inclusive participation, enhancing board diversity, and implementing robust accountability measures to improve governance practices.

Beyond these empirical contributions, the study underscores that governance in sport is ultimately a human practice. Leadership and management within tennis clubs are not solely matters of structure or regulation, but of relationships built on trust, dialogue, and shared responsibility. Effective governance emerges when participation becomes voice, accountability becomes care, and leadership becomes service. In this sense, good governance is less a system of control than a continuous conversation—an ethical and participatory process through which clubs nurture both performance and community.

This section includes the main limitations, future research directions, and practical applications of the study.

### Limitations

Despite adhering to established research methodologies commonly employed in similar studies, this research has several limitations that warrant consideration.

Firstly, the sample size comprises 30 tennis clubs, which, while substantial, may not fully capture the diversity of experiences and practices across a broader spectrum of clubs. This reduced sample constrains the robustness of statistical interpretations and limits representativeness across the wider tennis ecosystem. A larger sample size could potentially yield different insights and enhance the robustness of the findings.

Secondly, the geographical distribution of the clubs involved in the study is limited and restricts the generalizability of the findings, and thus the extrapolation should not go beyond the findings. The inclusion of clubs from additional countries, especially those in diverse tennis regions and with varied cultural contexts, could present alternate scenarios and contribute to a more comprehensive understanding of the subject matter.

Thirdly, the characteristics of the clubs, including whether they are private or public, may influence the findings. The study does not account for potential variations in organizational practices and perspectives between different types of clubs, which could affect the generalizability of the results.

Lastly, the questionnaire utilized in this study, although carefully designed, has its constraints. The inclusion of more questions or different types of questions might have captured a wider array of data and provided a more nuanced view of the clubs' perspectives.

In conclusion, while the results of this study should be interpreted with caution and not be generalized across all tennis clubs, they offer valuable insights into the views and practices of the participating organizations. These findings contribute to the existing body of knowledge and can serve as a foundation for future research that addresses these limitations.

### Future research directions

Future research should explore how these metrics interact over time and investigate strategies for promoting gender equality as a core component of governance across diverse organizational contexts. It should aim to include larger and more diverse samples of tennis clubs from other cultural and organizational contexts, enabling comparative and longitudinal analyses across regions and time periods.

The present study opens several avenues for future research that can deepen the understanding of governance in tennis clubs and explore the nuances across different contexts and variables.

Firstly, subsequent studies could expand the geographical scope by involving tennis clubs from various regions and continents. Such research would be instrumental in identifying and analysing cultural differences in governance practices, potentially revealing diverse approaches and solutions that can be applied in different cultural contexts.

Secondly, an important research direction could be the examination of governance differences among various types of clubs, such as private, public, and commercial entities. Understanding how governance structures and challenges vary across these categories can provide valuable insights for tailored governance strategies.

Additionally, future research could explore the implications of governance aspects in relation to the geographic location of the clubs. This includes urban vs. rural settings and regions with varying levels of sports infrastructure and development. Such studies can illuminate how location-specific factors influence governance practices and outcomes.

Another potential area of study is the socio-economic characteristics of club members. Researching how different socio-economic backgrounds affect governance preferences and effectiveness could provide targeted recommendations for clubs with diverse member bases.

The degree of public or government assistance available to clubs is another crucial variable that warrants further exploration. Studies focusing on how external support impacts governance can guide policy decisions and the allocation of resources to optimize club governance.

Moreover, future research could explore the role of regulatory frameworks and funding mechanisms in shaping governance outcomes, offering a pathway for continued improvement and innovation in sports governance.

Finally, future research could examine the role of sponsorship opportunities in shaping governance practices. Understanding the relationship between access to sponsorship and governance quality can help clubs leverage external funding to improve their management and operations.

In conclusion, these future research directions promise to enrich the body of knowledge on tennis club governance, offering practical insights and guidance for a wide range of stakeholders.

### Practical applications

The cluster-based analysis provides actionable insights for governance improvement. Clubs in Cluster 1 could benefit from increasing their transparency and independent board representation, while those in Cluster 2 should focus on stabilizing leadership despite their strengths in transparency. Cluster 3, with its balanced and engaged governance profile, can serve as a model for best practices. Finally, Cluster 4 requires comprehensive reforms to address deficiencies in participation, transparency, and independent oversight.

The comparative focus across Southern Europe is one of the study's most distinctive and valuable contributions. From a cultural narrative perspective, Southern European clubs often embody a hybrid form of governance, grounded in volunteerism, family ties, and historical identity, yet increasingly shaped by modern expectations of professionalism and accountability. It is then relevant to highlight this tension—between the intimacy of community and the structure of modern management—as it is believed that it emphasises the paper's originality.

The findings of this study, even though should be carefully considered to avoid extrapolations that do not go beyond the findings, have significant practical applications for various stakeholders within the tennis community. The results can be instrumental in informing and improving governance practices among tennis clubs, thereby enhancing their overall functioning and sustainability.

For club volunteers and managers, this research provides a deeper understanding of the governance challenges faced by tennis clubs. By highlighting specific areas of concern and potential improvement, the study equips these stakeholders with the knowledge necessary to implement more effective governance strategies and practices.

Club members can benefit from this study by gaining a clearer picture of the primary governance aspects that influence their clubs. An informed membership is better positioned to engage in club activities and decision-making processes, contributing to a more transparent and democratic governance structure.

National and regional federation staff and board members can utilize the insights from this research to tailor their support and assistance programs more effectively. Understanding the governance issues at the club level allows these governing bodies to provide targeted resources and interventions that address the specific needs of their member clubs.

Researchers in the field of sports management and governance will find the study's results valuable for advancing their work. The identified governance issues and trends can serve as a foundation for further research, opening up new lines of inquiry and contributing to the development of best practices in club governance.

In summary, the practical applications of this study are far-reaching, offering valuable guidance and support to tennis club stakeholders, federation officials, and academic researchers. By fostering a better understanding of governance issues, this research contributes to the overall enhancement of governance practices within the tennis community. In this context, leadership and governance in tennis clubs should be viewed as participatory, ethical, and dialogical processes grounded in trust, transparency, and collaborative engagement among stakeholders. Good governance begins when management becomes dialogue, and leadership becomes service.

Our research transcends into a clearer emotional and ethical resonance—the recognition that sports governance, at its heart, is a human relationship. To govern well is to care for others, to create trust, and to hold responsibility not only as a duty, but as an act of shared learning. That is where the science of governance meets the soul of sport.

## Data Availability

The raw data supporting the conclusions of this article will be made available by the authors, without undue reservation.
